# Characterization of anti-EBA175RIII-V in asymptomatic adults and children living in communities in the Greater Accra Region of Ghana with varying malaria transmission intensities

**DOI:** 10.1186/s12865-018-0271-y

**Published:** 2018-11-19

**Authors:** L. E. Amoah, H. B. Abagna, K. Akyea-Mensah, A. C. Lo, K. A. Kusi, B. A. Gyan

**Affiliations:** 1 Noguchi Memorial Institute for Medical Research, University of Ghana, Accra, Ghana; 2 Present address: University Cheikh Anta DIOP, Dakar, Senegal

## Abstract

**Background:**

Antibodies against Region III-V of the erythrocyte binding antigen (EBA) 175 (EBA175RIII-V) have been suggested to provide protection from malaria in a natural infection. However, the quality and quantity of naturally induced antibodies to EBA175RIII-V has not been fully characterized in different cohorts of Ghanaians. This study sought to determine the characteristics of antibodies against EBA175RIII-V in asymptomatic adults and children living in two communities of varying *P. falciparum* parasite prevalence in southern Ghana.

**Methods:**

Microscopic evaluation of thick and thin blood smears was used to identify asymptomatic *Plasmodium falciparum* carriage and indirect enzyme linked immunosorbent (ELISA) used to assess antibody concentrations and avidity.

**Results:**

Parasite carriage estimated by microscopy in Obom was 35.6% as opposed to 3.5% in Asutsuare. Levels of IgG, IgG1, IgG2, IgG3 and IgG4 against EBA175RIII-V in the participants from Obom were significantly higher (*P* < 0.05, Dunn’s Multiple Comparison test) than those in Asutsuare. However the relative avidity of IgG antibodies against EBA175RIII-V was significantly higher (*P* < 0.0001, Mann Whitney test) in Asutsuare than in Obom.

**Conclusions:**

People living in communities with limited exposure to *P. falciparum* parasites have low quantities of high avidity antibodies against EBA175RIII-V whilst people living in communities with high exposure to the parasites have high quantities of age-dependent but low avidity antibodies against EBA175RIII-V.

**Electronic supplementary material:**

The online version of this article (10.1186/s12865-018-0271-y) contains supplementary material, which is available to authorized users.

## Introduction

The asexual stages of *Plasmodium falciparum* (*P. falciparum*) are partly responsible for the pathology associated with malaria and subsequently are the focus of malaria treatment regimens as well as the focus of malaria vaccine research. The merozoite is the only extracellular stage of the parasites erythrocytic life-cycle, making merozoite surface antigens promising malaria vaccine candidates. One such candidate is the erythrocyte binding antigen (EBA) 175 (EBA 175, Pf3D7_0731500), PfEBA-175 has been shown to play a key role during the fast cascade of interactions between the parasite and host molecules before the merozoite completely invades the erythrocyte by binding to sialic acid residues on glycophorin A on the red blood cell during merozoite invasion [1]. Of the 6 extracellular domains of EBA175, region 2 (RII), which comprises of two cysteine rich domains F1 and F2 [2] as well as RIII-V, which comprises of the dimorphic region 3 (RIII) as well as the highly conserved regions 4 (RIV) and 5 (RV) [2] have been implicated as vaccine candidate antigens.

Antibodies induced against diverse antigenic components of the erythrocytic parasite are important mediators of anti-disease immunity [3]. Some known functions of antibodies induced against the asexual parasite include preventing merozoites from invading new erythrocytes (inhibition of invasion), preventing cytoadherence of infected erythrocytes to endothelial cells as well as interfering with the normal function of monocytes and macrophages [4, 5]. Targeting the merozoite before they invade erythrocytes can serve as a means to truncate the infection. The ability of antibodies against the merozoite to prevent erythrocyte invasion has been demonstrated through in vivo human passive transfer assays [6, 7] and in vitro erythrocyte invasion inhibition assays [8] several years ago. Antibodies specific for EBA175 RIII-V have been shown to be associated with protection from malaria in symptomatic cases [9]. Also, Healer et al., [10] in immunization studies have shown that antibodies induced by a recombinant RIII-V inhibit merozoites invasion.

Repeated exposure to malaria parasites has been suggested as a necessary requirement for the maintenance of anti-parasite immunity as it has been demonstrated in a number of studies that antibodies against merozoite antigens are relatively short-lived in the absence of a new infections [11–14]. This has been confirmed in some community studies where people with current infections had higher merozoite antibody levels than those without [15–17]. However, a few studies including one by Wipasa et al., noticed that both antibody and memory B cell responses to malaria antigens remained steady over long periods in the absence of an infection [18]. Some studies have suggested that merozoite antibody levels show a direct correlation with malaria transmission intensity in malaria endemic settings and are higher in high transmission settings [19, 20].

The cytophilic immunoglobulins, IgG1 and IgG3 have been associated with parasite repression directly, or opsonization indirectly [21]. IgG1 and IgG3 antibodies to merozoite antigens generally have short half-lives [12]. The half-lives of IgG subclass responses against EBA175 are generally short lived, however, the half-lives of IgG1 and IgG3 have been noted to be longer lived and more prevalent than those of IgG2 and IgG4 [21, 22]. IgG1 and IgG3 responses against EBA175 have also been associated with lower parasitaemia in a high transmission setting [21] as well as a seasonal transmission setting of Papua New Guinea [23].

The process of antibody selection that occurs during humoral immune response maturation, results in the production of antibodies with increased avidity [24, 25]. Antibody properties, including high avidity, have been suggested to key determinants of protective immunity against malaria [26–28]. High Avidity to whole schizont extract as well as to a number of specific *P. falciparum* antigens, have been shown to correlate with protection from malaria [29–31]. The avidity of antibodies against MSP1 has been observed to increase after a recent *P. falciparum* infection [29], however some reports have implicated reduced antibody affinity maturation and antibody avidity to a recent malaria infection [32] and excessive stimulation of B cells in high parasite prevalence settings [33]. The avidity of antibodies to *P. falciparum* antigens has been found to be lower in areas of high malaria transmission intensity than in areas with lower transmission [34].

This study sought to determine differences in the characteristics of antibody responses to EBA175RIII-V in adults and children living in high and low malaria parasite prevalence settings.

## Methods

### Ethical consideration

Ethical approval for the study (#089/14–15) was obtained from the Institutional Review Board of the Noguchi Memorial Institute for Medical Research. Written informed consent, assent and parental consent were obtained for all participants recruited into the study.

### Study site and population

**Fig. 1:**
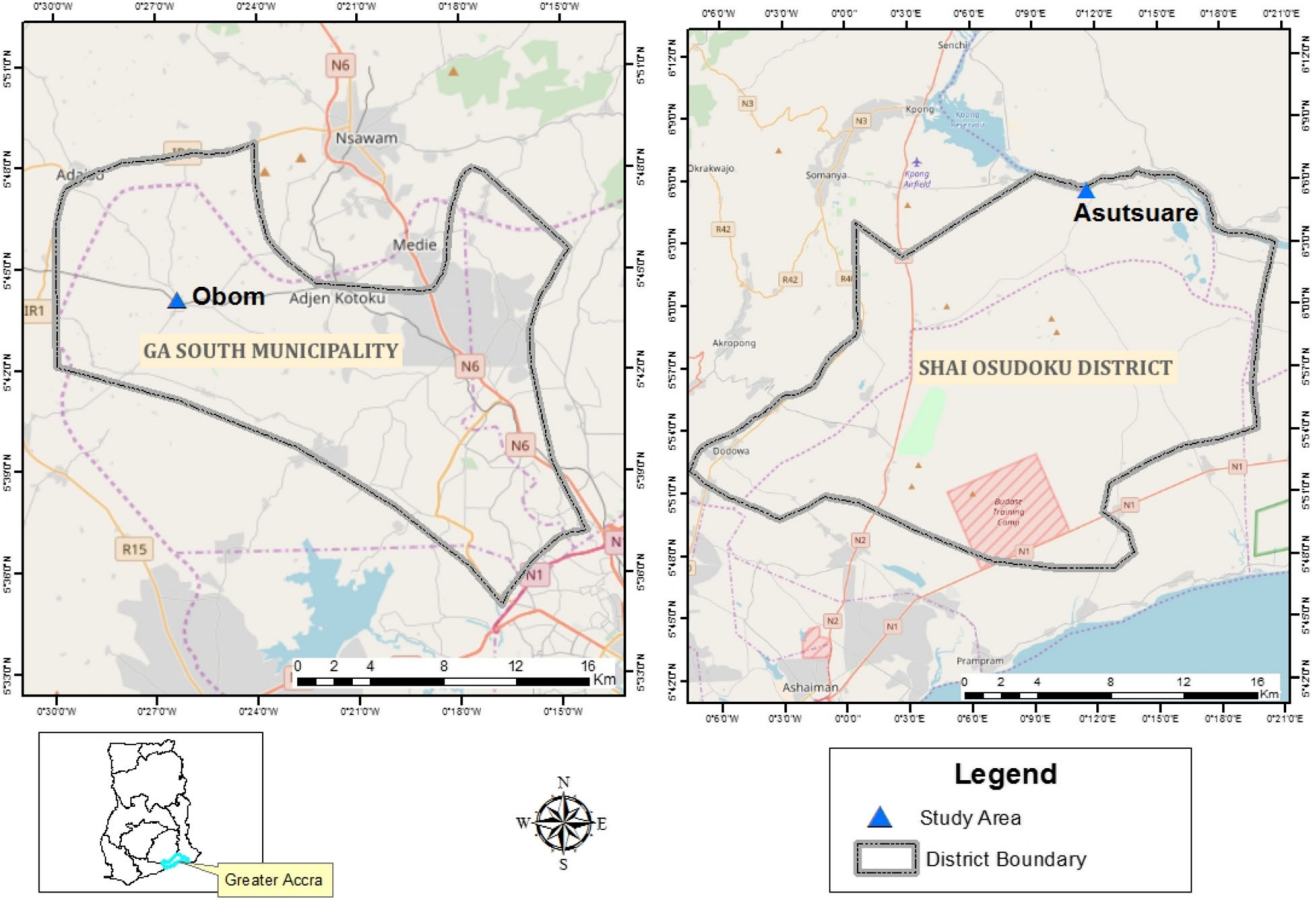
Map of Ghana highlighting study sites. A map of Ghana, highlighting the Greater Accra Region where the two sites are located and including a detailed presentation of both study sites was created by Mr. Richard Adade using shapefiles and ArcMap GIS v10.5. No permission was required to access the shapefiles from the Survey Department of the Ghana Statistical Services

The cross-sectional study conducted in June 2016, recruited adults and children aged between 2 and 75 years from two semi-rural communities, Obom and Asutsuare, both within the Greater Accra Region of Ghana as part of a large study which aims to identify a number of factors that influence asymptomatic *P. falciparum* carriage in high and low malaria transmission settings in Ghana. This study only recruited people in the two communities who did not exhibit any sign or symptom of clinical malaria and provided written informed consent for either themself or a dependent. Obom is a high *P. falciparum* prevalence community in the Ga South Municipality and Asutsuare is a low *P. falciparum* prevalence community, with noted low malaria transmission [35] in the Shai Osudoku District (Fig. 1). The major malaria season in the Greater Accra Region is from June to August, with a peak in July [36].

### Sample collection and processing

After obtaining written informed consent, 5 ml of venous blood was collected from each participant into acid citrate dextrose (ACD) vacutainer tubes. A drop of the whole blood was used to prepare thick and thin blood smears and the rest was separated into plasma and packed cells after centrifugation. The plasma was immediately stored at -20 °C. Demographic data from the participants including ownership of insecticide treated bed nets (ITN) was also captured.

### Microscopic identification of *P. falciparum* parasites

Thin and thick blood smears were processed using a method described by the WHO [37]. Briefly thin blood smears were dried, fixed in 100% methanol and then stained with 10% Giemsa after the methanol had evaporated. Thick blood smears were air-dried and stained with 10% Giemsa. The thick and thin smears were observed under an × 100 oil immersion objective by two independent microscopists. The thin smears were used to identify the infecting *Plasmodium* species [38].

### Enzyme linked immunosorbent assay (ELISA)

*Lactococcus lactis* produced EBA175-RIII-V [39] was used in an indirect ELISA to measure total IgG and IgG subclass (IgG1, IgG2, IgG3 and IgG4) antibody responses in plasma from the study participants using a protocol similar to that previously reported by Acquah, F et al [39] for IgG and a modification of Ismail, HA et al [21] for the IgG subclasses. Briefly, 1 ng of purified antigen, EBA175-RIII-V in phosphate buffered saline (PBS, pH 7.4) was coated 100 μl /well onto NUNC MaxiSorp™ ELISA plates (Thermo Scientific, UK) overnight at 4 °C. Plates were blocked with 150 μl/well of 3% (*w*/*v*) skimmed milk powder (Marvel, UK) in PBS/T after four washes using PBS supplemented with 0.05% Tween 20 (PBS/T). Duplicate wells of the plates were then incubated with 100 μl of plasma diluted 200-fold and a reference standard, purified human polyclonal IgG [40, 41] (BP055, The Binding Site, UK) at a starting concentration of 1000 ng/μl and serially diluted 3-fold in duplicate wells was used as a standard calibrator.

Plates were incubated for an hour at room temperature and then washed four times with PBS/T. The plates were subsequently incubated with 50 μl of goat anti-human IgG-HRP (Invitrogen, USA) secondary antibodies for IgG.

For the IgG subclass ELISA, the plates were processed as for the IgG above, however the plates were incubated with 100 μl of plasma diluted 1:50 at 37 °C for 1 h and after washing, incubated with 50 μl of goat anti-human IgG1-HRP (The Binding Site, UK) and goat anti-human IgG3-HRP (The Binding Site, UK) secondary antibodies. A positive control sample was obtained by pooling a number of samples that had previously identified as containing high concentrations of EBA175-RIII-V [42]. This positive control sample was serially diluted to prepare the standard curve) at a starting concentration of 1:10 and serially diluted 2 fold. Sera from adults living in the USA who have never been exposed to malaria (malaria naïve sera) and confirmed as having extremely low concentrations of EBA175RIII-V antibody levels were used as negative control samples. A positive and some negative control samples were used on each ELISA plate.

All plates were developed by adding 50 μl/well of 3,3′,5,5′-tetramethylbenzidine (TMB) solution for 15 min for total IgG or 20 min for the IgG subclasses and then stopped with 2 M H_2_SO_4_. Fluorescence was measured immediately after stopping the reactions using excitation wavelength of 450 nm.

### IgG avidity ELISA

A procedure similar to the total IgG ELISA described above was used, however four replica wells were incubated with each appropriately diluted plasma sample for an hour, after which 100 μl/well of 2.4 M sodium thiocyanate (NaSCN; Sigma-Aldrich, UK) was added for an extra 10 min incubation [42–44] prior to the wash and subsequent addition of 50 μl/well of goat anti-human IgG-HRP (Invitrogen, USA).

### Data analysis

A thick blood smear was classified as negative for *Plasmodium* parasites if no infected erythrocytes were observed after counting 200 WBCs by both microscopists. In instances where disparities were observed in identifying the presence of *Plasmodium* infected erythrocytes, the smear was given to a third microscopist to confirm the presence or absence of *Plasmodium* infected erythrocytes. Once a thick smear was identified as containing *Plasmodium* infected erythrocytes, the corresponding thin smear was inspected to determine the *Plasmodium* species present in the sample.

Demographic data was entered into excel and column statistics determined using GraphPad Prism v5 (GraphPad software, USA).

Optical density (OD) results from the ELISA plate reader were converted into concentrations using the four-parameter curve-fitting program known as ADAMSEL (Ed Remarque, BPRC) and the data analyzed using GraphPad Prism v5 (GraphPad software, USA). The correlation between age and EBA175RIII-V antibody (IgG, IgM, IgG1 and IgG3) concentrations were performed using Spearman non-parametric correlation matrix and Mann Whitney U tests used to determine the differences in similar antibody responses between the two sites.

Seropositivity was defined as antibody concentration higher than the average antibody concentration of the negative control samples (naïve serum) plus two standard deviations.

Relative antibody avidity was determined as the ratio of the mean IgG concentration of the SCN^−^-treated sample to the mean IgG concentration of the untreated sample multiplied by 100. (Avidity index = [antibodies following NaSCN treatment/ antibodies without NaSCN treatment] × 100).

Participants were stratified into three age groups, ≤10 (ten and below 10), 11–14 and ≥ 15 (fifteen and above) for some of the analysis. Data from seven participants from Asutsuare were excluded in age stratified analysis because their ages were not recorded. Statistical significance was defined as *P* value ≤0.05 unless otherwise stated.

## Results

The age range for the 161 study participants from Obom was 6 to 70 years, while that of the 169 participants from Asutsuare was 2 and 75 years (Table 1). Asymptomatic *P. falciparum* parasite carriage, as determined by light microscopy, was higher in Obom (57 of the 160 participants or 35.6%) compared to Asutsuare (6 of the 169 participants, representing or 3.5%). There was thus a 10-fold difference in parasite carriage between the two study communities (Table 1). Bed net ownership was low in both communities, only 11 and 2 participants in Obom and Asutsuare respectively claimed to own bed nets.

**Table 1:** Characteristics of study participants

	*N*	% Asymptomatic infections	Median age (years)
Obom	161	35.6	15 (6–70)
Asutsuare	168	3.5	16 (2–75)

*N* total number of volunteers enrolled, % Asymptomatic is the % of people that tested positive for *P. falciparum* by microscopy. Median values reported with minimum and maximum age values

### Seroprevalence of antibodies against recombinant EBA175RIII-V_Ll_ antigen

The cutoff values used to calculate seroprevalence was 2581, 1773, 580 and 1198 AU for IgG1, IgG2, IgG3 and IgG4 respectively and 2137 ng/ml for IgG. The seroprevalence of IgG and IgG subclasses to EBA175RIII-V_Ll_ of participants from Obom (IgG, 85.6%; IgG1, 90.6%; IgG2, 51.3%; IgG3, 91.3% and IgG4, 25.0%) was significantly higher (*P* < 0.05, Mann Whitney test) than participants from Asutsuare (IgG, 58.0%; IgG1, 34.9%; IgG2, 5.9%; IgG3, 31.4% and IgG4, 16.0%). Seropositivity to the cytophilic IgG subclasses, IgG1 and IgG3 against EBA175 RIII-V_Ll_ in both Obom and Asutsuare was higher than seropositivity to IgG2 and IgG4.

### Concentration of antibodies against recombinant EBA175RIII-V_Ll_ antigen

Although 161 and 169 participant samples were used for all the different ELISAs, some samples had values, which were classified as ‘Low’ by the plate reader, meaning their value was similar to the blank sample and were not assigned a value. Those samples were not included in the analysis and subsequently resulted in variations in the final total number of samples, N used in the analysis.

**Fig. 2:**
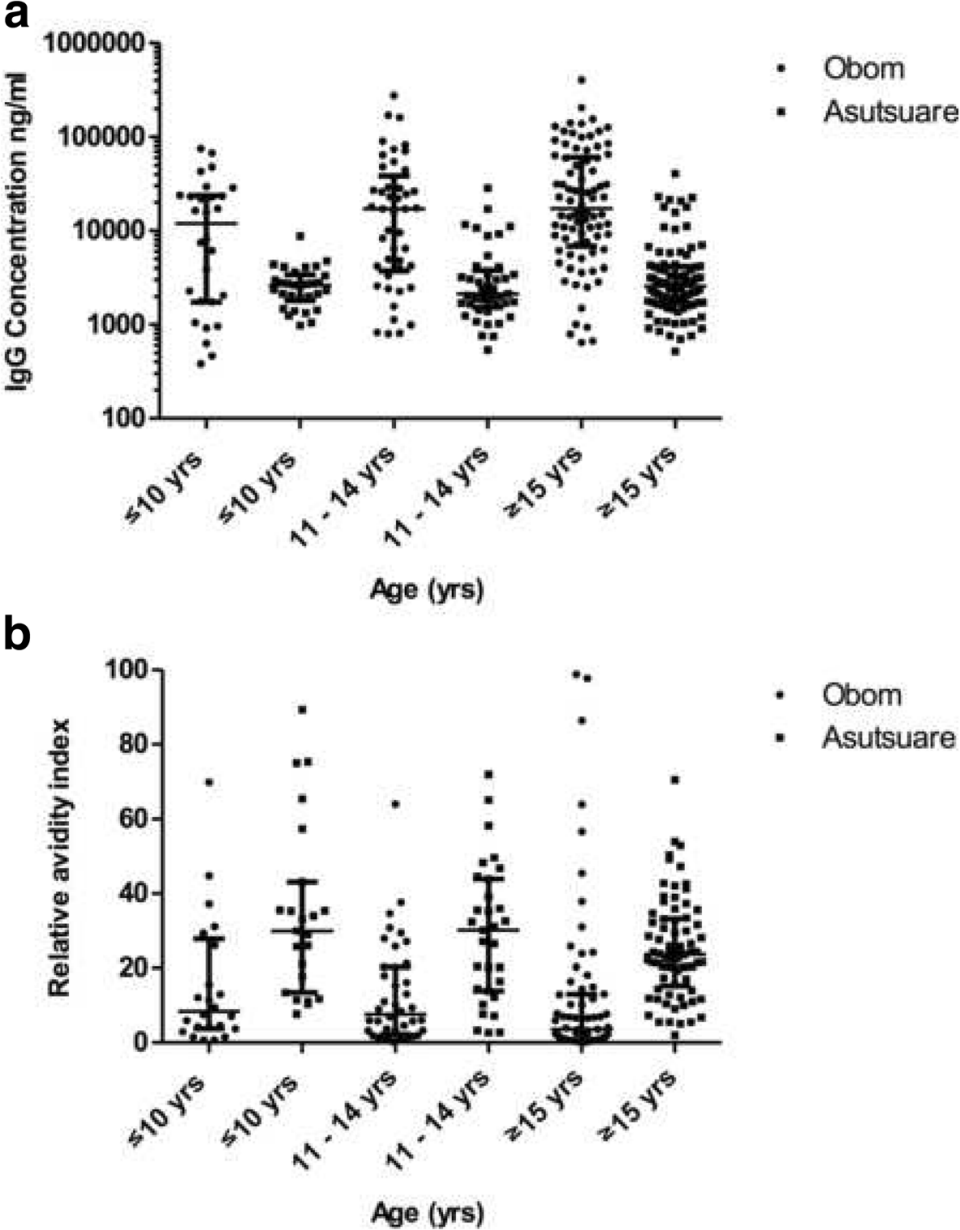
Age stratified IgG concentrations and avidity. Participants in Obom (black circles) and Asutsuare (black squares) were stratified into three age groups, ≤10, 11–14 and ≤ 15 years. The concentrations (**a**) and relative avidities (**b**) of naturally induced IgG antibodies against EBA175RIII-V antigen in plasma samples obtained from whole blood collected in June 2016 was measured using ELISA as described in the methods section. The graphs represent the median concentrations with the interquartile range as the error bars

**Fig. 3:**
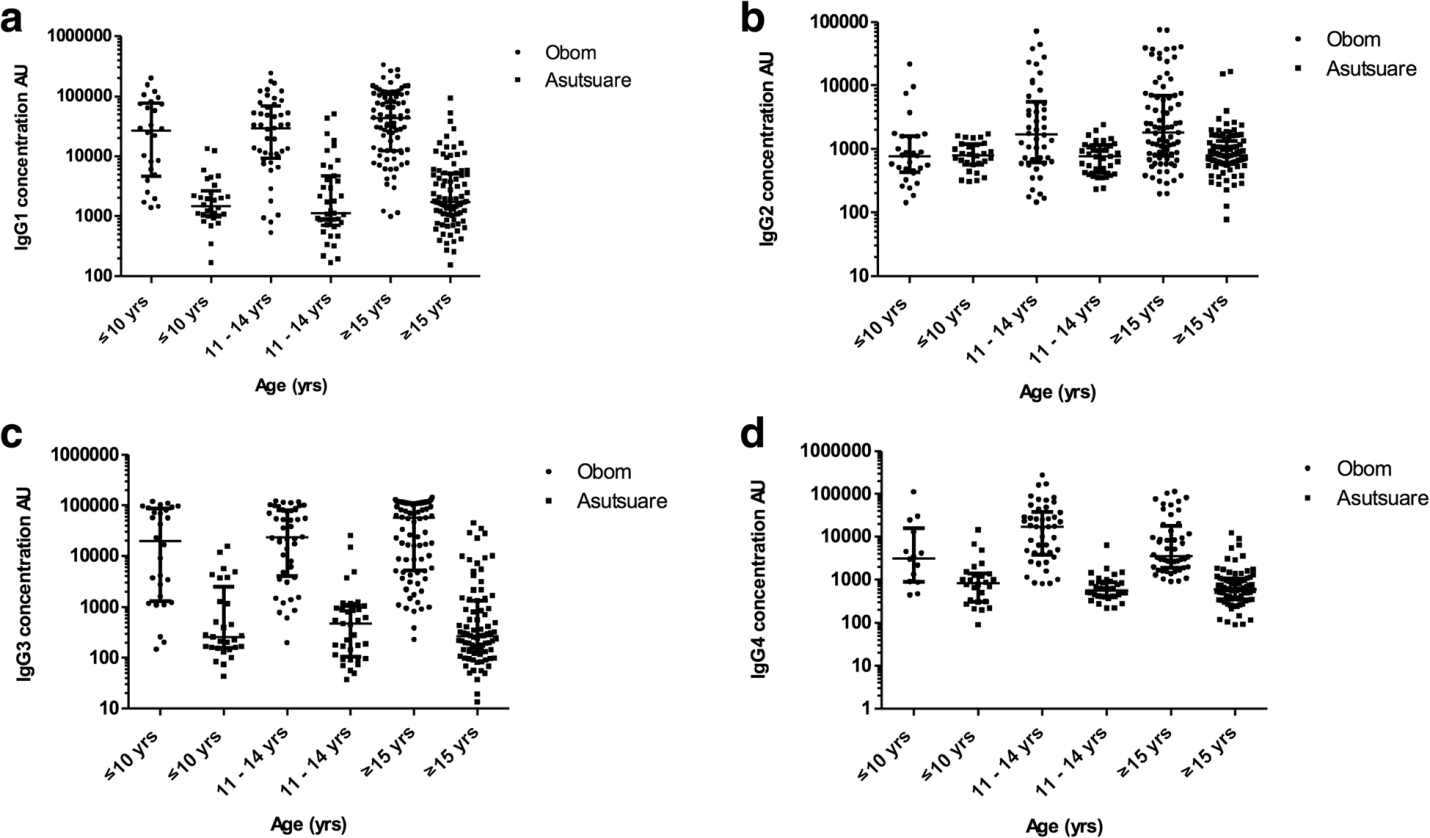
Antibody subclass responses. An ELISA protocol similar to that used to produce the graphs in Fig. 2 was used to determine the concentration of naturally induced IgG1 (**a**), IgG2 (**b**), IgG3 (**c**) and IgG4 (**g**) levels in plasma samples obtained from whole blood collected from the study participants. The graphs represent the median concentrations with the interquartile range as the error bars

The median antibody concentrations for both IgG and all the four IgG subclasses (IgG1, IgG2, IgG3 and IgG4) measured in participants from Obom were significantly higher than those recorded for from participants from Asutsuare (*p* < 0.0001, Mann Whitney test for each) (Figs. 2a and 3a-d). The cytophilic IgG1 and IgG3 antibody responses measured in both sites were higher than the antibody concentrations of IgG2 and IgG4 (Table 2).

**Table 2:** IgG and IgG subclass antibody concentrations

		N	Min	Median	Max
IgG	Obom	156	380.5	16,006	403,567
(ng/ml)	Asutsuare	168	518.8	2411	40,686
IgG1	Obom	153	526.8	33,041	331,686
(AU)	Asutsuare	165	154.2	1660	93,998
IgG2	Obom	158	142.2	1409	75,188
(AU)	Asutsuare	162	76.67	770	16,397
IgG3	Obom	156	146.8	40,009	141,652
(AU)	Asutsuare	156	13.4	270.7	45,058
IgG4	Obom	88	76.98	3152	113,147
(AU)	Asutsuare	143	89.89	605.8	14,617

*N* total number of samples used in the analysis, *Min* minimum concentration, *Max* maximum concentration. Total IgG was measured in ng/ml, whilst the IgG subclasses were measured in arbitrary units (AU)

Levels of IgG, IgG1, IgG2 and IgG3 antibody responses significantly correlated with age in Obom (Spearman rho: 0.2244, 0.2677, 0.2210 and 0.1724 respectively; *P* < 0.05 in all cases). However, there was no correlation between the levels of IgG4 antibody responses in Obom or any of the antibody responses measured in samples from Asutsuare with age (Fig. 3).

### Antibody avidity

The relative avidity index of IgG responses measured in participants from Obom and Asutuare both significantly correlated negatively with age, (Spearman *r* = − 0.2338, *p* < 0.0072 in Obom; Spearman *r* = − 0.1824, *p* = 0.0394 in Asutsuare). Although the antibody concentrations measured in volunteers from Asutsuare were significantly lower than those measured in volunteers from Obom (Fig. 2a), the relative avidities of IgG antibodies against EBA175RIII-V were higher in the volunteers from Asutsuare than from Obom (Fig. 2b). Antibody avidity index (RAI) of IgG for participants from Obom who were 10 years old and below were significantly lower (*p* < 0.05, Dunn’s multiple comparison test) than the RAI for all three categories (10 years and below, between 11 and 14 years and those 15 years and above) of participants in Asutsuare (Additional file 1). The RAI of IgG for participants from Obomaged 10 years and below was significantly higher (*p* < 0.001, Dunn’s multiple comparison test) than participants aged between 11 and 14 years as well as those 15 years and above (Additional file 1).

## Discussion

Rabbit serum containing polyclonal antibodies against Region III-V of EBA175 have been found to directly inhibit *P. falciparum* merozoite invasion [10]. Naturally induced antibodies against Region III-V of EBA175 have also been suggested to be indicative of strong protection from symptomatic malaria [45], however the role antibodies against EBA175RIII-V play in asymptomatic malaria has not been fully evaluated. Data from the assessment of the magnitude and avidity of antibody responses to this antigen across different age groups will potentially be relevant for the interpretation of natural and vaccine induced immune responses to this antigen.

In order to determine possible differences in natural antibody responses to Region III-V of EBA175, a cross sectional survey was carried out in Obom, which has been reported to be a high parasite prevalence community [46] and in Asutsuare, where parasite prevalence and malaria transmission intensity is known to be very low [35]. The study enrolled a range of children and adults to determine possible differences in age related antibody responses. This study confirmed the existence of very low *P. falciparum* parasite prevalence in Asutsuare (Table 1) as has previously been reported [35]. The number of asymptomatic individuals in Asutsuare however may be higher than the 3.5% if *Plasmodium* parasites were detected by molecular methods. Especially as a higher number of submicroscopic parasite infections are detected in asymptomatic individuals due to the increased sensitivity of the detection technique [47]. Bed net ownership was generally low in both communities. Ownership in Obom was higher than in Asutsuare, most likely because residents of Asutsuare encounter very low frequencies of malaria that they no longer think it is important to implement personal malaria control interventions relative to inhabitants of Obom, where malaria transmission is high.

Although only 3.5% of the participants from Asutsuare had microscopic densities of parasites, almost 60% of them were seropositive for anti-EBA175RIII-V antibodies. Similarly, although only about 35% of the participants from Obom were identified by microscopy as harboring *P. falciparum* parasites, over 80% had IgG antibodies against EBA175RIII-V. These collectively suggest the possible presence of parasites at densities below the detection limit of microscopy in some of the participants or that some of the participants had just recently cleared a *P. falciparum* infection, as antibodies against EBA175 have been suggested to be relatively short-lived [21].

The significantly higher IgG responses recorded in Obom than in Asutsuare (Fig. 2a) is indicative of the possible requirement of an active infection to induce antibodies against EBA175RIII-V as has been previously suggested, especially as these antibodies are relatively short-lived [21]. IgG subclass responses in both sites were predominantly cytophilic (IgG1 and IgG3) (Fig. 3), which supports a number of previous reports [9, 21, 48, 49]. However responses to IgG2 and IgG4 existed, although at much lower quantities and has been suggested to be due to the much shorter half-lives of IgG2 and IgG4 compared to IgG1 and IgG3 [21]. IgG subclass antibody levels were significantly higher in Obom than in Asutsuare, which was not surprising as total IgG levels were also significantly higher in Obom than in Asutsuare.

In Obom, the high transmission setting, IgG, IgG1 and IgG3 responses to EBA175RIII-V positively correlated with age (Figs. 2a, 3a&c). This supports data from a previous study conducted in a high transmission setting in Nigeria [21] and that of another study in highly asymptomatic children from a moderately seasonal setting Papua New Guinea [9]. High IgG1 and IgG3 responses for other merozoite antigens such as MSP1 [50] and MSP2 [51] have also been documented. A previous study did not find any correlation between IgG2 and IgG4 responses against EBA175 and age [21], however in this study, IgG2 responses correlated positively (Fig. 3b) with age in responses measured in the high transmission setting, Obom. The lack of age associated antibody responses in Asutsuare (Figs. 2a & 3) may be due to the very low prevalence of parasites observed in the community (Table 1) as parasite exposure has been found to be necessary for mounting immune responses against malaria antigens.

The relative antibody avidities measured in the participants from Asutsuare, the low transmission setting were significantly higher than those measured in Obom (Fig. 2b). A similar observation has been reported for antibody responses to this same antigen conducted in children living in the same high transmission setting and a different low transmission setting in Ghana [42] as well as for antibody responses to a different merozoite antigen, MSP1_19_ [34] and could be due to the presence of fewer and less diverse parasite clones circulating in low transmission zones [52]. Although high parasite diversity and frequency of infection is anticipated in high transmission settings, the diversity of RIII-V of EBA175 was recently suggested to be relatively similar in parasites circulating in Obom (the high transmission setting) and Abura [42], a community with low parasite transmission intensity, similar to Asutsuare. Generally, an increase in exposure to diverse parasites strains/isolates will result in heterologous exposure, which could lead to reduced affinity maturation and the production of antibodies with lower avidities as measured in antibody responses in Obom. A recent report on the avidity of naturally induced antibodies against EBA175RIII-V in children living in southern Ghana similarly reported reduced avidity of antibodies from children living in the high transmission setting compared to the low transmission setting [42].

The likely short longevity of naturally induced antibodies against EBA175RIII-V is contrary to the dynamics of antibodies against MSP1–_19_, which have been found to persist for several years after the clearance of *P. falciparum* parasites [53, 54] and thus may be a suitable candidate to use as a serological marker to monitor changes in malaria transmission intensity.

## Conclusion

People living in communities with limited exposure to *P. falciparum* parasites have low quantities of high avidity antibodies against EBA175RIII-V whilst people living in communities with high exposure to the parasites have high quantities of age dependent but low avidity antibodies against EBA175RIII-V.

## Additional file

Additional file 1: A tabular (a) and the corresponding column statistics (b) obtained from a One way ANOVA analysis (Prism v5) of the relative avidity of naturally induced antibodies against EBA175RIII-V in the age stratified ≤10, 11–14 and ≥ 15 years participants from Obom and Asutsuare. (DOCX 50 kb)

## Abbreviations

ACD: Acid citrate dextrose
AU: Antibody unit
DBS: Dried blood spot
EBA 175: Erythrocyte binding antigen 175
ELISA: Enzyme linked immunosorbent assay
IgG: Immunoglobulin
OD: Optical density
