# Supplementary material for: Characterization of anti-EBA175RIII-V in asymptomatic adults and children living in communities in the Greater Accra Region of Ghana with varying malaria transmission intensities
Source: BMC Immunol. 2018 Nov 19;19:34. doi: 10.1186/s12865-018-0271-y (PMC6245760; doi:10.1186/s12865-018-0271-y)
Supplement: Supplementary file 1 — A tabular (a) and the corresponding column statistics (b) obtained from a One way ANOVA analysis (Prism v5) of the relative avidity of naturally induced antibodies against EBA175RIII-V in the age stratified ≤10, 11–14 and ≥ 15 years participants from Obom and Asutsuare. (DOCX 50 kb) [file 12865_2018_271_MOESM1_ESM.docx]

Additional file 1. Statistical analysis of the relative avidity of IgG responses

A tabular (a) and the corresponding column statistics (b) obtained from a One way ANOVA analysis (Prism v5) of the relative avidity of naturally induced antibodies against EBA175RIII-V in the age stratified ≤10 years (10 years and below), 11-14 years and ≥15 years (15 years and above) participants from Obom and Asutsuare
